# The relationship between thyroid function tests and sleep quality: cross-sectional study

**DOI:** 10.5935/1984-0063.20200050

**Published:** 2021

**Authors:** Mohammad Reza Nazem, Ehsan Bastanhagh, Ali Emami, Mehdi Hedayati, Saghar Samimi, Masoumeh Karami

**Affiliations:** 1 Department of Clinical Biochemistry, Faculty of Medical Sciences, Tarbiat Modares University, Tehran, Iran.; 2 Department of Anesthesiology, Pain and Critical Care, Yas Hospital, Tehran University of Medical Sciences, Tehran, Iran.; 3 Cellular and Molecular Endocrine Research Center, Research Institute for Endocrine Sciences, Shahid Beheshti University of Medical Sciences, Tehran, Iran.; 4 Department of Biochemistry, Medical Faculty, AJA University of Medical Sciences, Tehran, Iran.

**Keywords:** Sleep Deprivation, Triiodothyronine, Thyroxine, Pittsburgh Sleep Quality Index, TSH, T_3_, T_4_

## Abstract

**Introduction:**

The relationship between sleep and hormones have long been recognized. Studies indicated that sleep quality is one of the major modulatory effects on the endocrine system. In this study, we aimed to assess the serum concentration of thyroid hormones in individuals who suffered from low quality sleep.

**Material and Methods:**

Based on the Pittsburgh Sleep Quality Index and ISMA Stress questionnaire, we divided 83 participants into two groups. Forty-one individuals with low quality sleep group and 42 with good quality sleep group, all from the male students of a medical school in Tehran, Iran, participated in this descriptive and cross-sectional study. Then, serum levels of thyroid hormones including free T_3_, free T_4_, and TSH were assessed and compared between two groups.

**Results:**

There were a significant increase in serum levels of FT_4_ (p=0.01) and TSH (p=0.02). There were also meaningful correlations between sleep score and stress score (p=0.008) as well as stress score and FT_4_ (p=0.03) in the case group.

**Conclusions:**

The current study showed that thyroid function tests (T_4_ and TSH) signiﬁcantly rose in the poor sleep condition. We also found correlations between sleep score, stress score, and FT_4_ in the poor sleep condition group that suggest low sleep quality can affect thyroid hormones.

## INTRODUCTION

Sleep has a key role to play in the maintenance of good health and wellbeing; which in turn leads to a better quality of life^1^. Individuals spend about one-third of their lives asleep^2^. It has been confirmed that abnormal sleep and some disorders link with thrombotic disease, epilepsy, arthritis, mood disorders, chronic pain, and diabetes mellitus^3-6^.

The quality and length of time asleep play a key role in sustaining normal function. It also supports the maintenance of daily metabolic and hormonal processes and helps with appetite regulation^7^. Some studies show that sleeping less than 6 hours a day is associated with disorders of energy metabolism^8^. This indicates that normal sleep is necessary for the regulation and release of thyroid hormones. Sleep restriction and disruption of an individual’s circadian clock are associated with other metabolic disorders such as obesity, insulin resistance, and diabetes^9^.

The relationship between sleep and hormones has long been recognized^10^. Studies indicate that deep sleep has a major modulatory effect on the endocrine system^11^. For example secretion of growth hormone (GH) and prolactin (PRL) is increased in deep sleep. Conversely, the release of cortisol and thyrotropin (TSH) is decreased. There is some evidence showing changes in the circadian rhythm, caused by sleep disruption and integrity, have an effect on the inflammatory system^12,13^.

Thyroid hormones, thyroxine (T4) and triiodothyronine (T3) exert physiologic change in all tissue metabolism. The secretion of T4 and T3 from the thyroid gland is controlled by the hypothalamic thyrotropin-releasing hormone (TRH) and the pituitary thyroid-stimulating hormone (TSH). TSH stimulates the thyroid gland to release thyroxine (T4). T4 is then converted to triiodothyronine (T3), which is the active hormone that stimulates human metabolism^14^. Sleep deviation can alter the function of the human hypothalamic-pituitary-thyroid axis; and is associated with altered levels of TSH, T4, and T3^15^. There is also a correlation between poor sleep quality and subclinical hypothyroidism^16^.

The reference interval for TSH varies significantly with age, sex, an hour of day, and ethnicity. TSH reference intervals are not affected by the time of year. Age, sex, an hour of day, and time of year do not affect the free T_4_ reference interval^17^. Despite the reduced serum T_4_ level in sleep-deprived rats, T3 levels are maintained. It may be the pituitary, which causes inappropriate TSH concentrations^18^. Sleep deprivation is very stressful for the individuals’ who suffer from it and it often gets disregarded by others. It is crucial to consider the role that the central nervous system (CNS) plays in this condition^19^. In this investigation, the aim is to show the effect of sleep quality and duration on thyroid hormone levels.

## MATERIAL AND METHODS

### Sample collection

In this study, eighty-three male students with an average age of 20 years (20.4 ± 1.1 years) took part. They were all assigned randomly into two groups. Forty-one individuals suffering from low quality sleep and 42 with good quality sleep. Participants were recruited from Medical School students in Tehran, Iran.

In order to eliminate any potential effect of sex and age on sleep duration, all participants of this study were male and in the same age group. The three main meals and the sleeping duration and conditions were the same for both groups. Everyone was woken up at the same time in the morning. The two groups were instructed to sleep between 22:00h and 05:30h. This regime was undertaken for six month before the study commenced and individuals were chosen whose daily routines were similar. No one in the group was taking any medication throughout this study.

The Pittsburgh Sleep Quality Index was used^20^ to identify individuals suffering from poor quality sleep. In 1988, PSQI was defined (Buysse et al.^20^) and the reliability and validity of it was confirmed through trails. The PSQI has seven components: subjective sleep quality, sleep latency, sleep duration, sleep efficiency, sleep disturbance, sleep medication use and daytime dysfunction and sleepiness. Each component has a range of 0 (no difficulty) to 3 (severe difficulty). The total score is between 0 and 21. A total score greater than 5 is associated with poor quality sleep.

To evaluate stress level, the ISMA questionnaire was used. It consists of 25 questions, each scoring one point. A score of 0-4 points indicates that the individual is unlikely to suffer from a stress-related illness. A score of 5-13 points shows a low level of stress; individuals in this group would potentially benefit from stress management therapy. Individuals’ scoring 14 points or more are suffering from significant stress, and require additional investigation and intervention through their medical practitioner. On this basis, forty-one individuals were grouped who suffered bad sleep and 42 individuals were grouped as they were assessed as having a good quality of sleep.

The study protocol was approved by the Ethical Committee of the University of Medical Science (number: 93-255/2015 March 10th) and with the 1964 Helsinki Declaration and its later amendments or comparable ethical standards. All protocols, guidelines and standard operating procedures were followed. Informed consent was obtained. There were no adverse incidents during this study.

### Biochemical assays

Blood samples were taken following aseptic techniques from the antecubital vein. The samples were taken at the same time in the morning (between 7:00 a.m. and 8:00 a.m.). Individuals fasted overnight and rested for a minimum of 30 min before the sample was taken. Infection prevention and blood storage guidance were complied with. The serum was isolated and stored at -80 ºC until assayed.

Serum values of FT3, FT4 and TSH were subsequently assessed. The level of three thyroid hormones (FT3, FT4 and TSH) were measured. Two (FT3 and FT4) were analyzed by commercially available electro-chemi-luminescence immunoassays (ECLIA) (Cobase 411, Roche Diagnostics GmbH, Mannheim, Germany). The sensitivity of the assays was 0.30pg/ml for FT3 and 0.40 for FT4ng/dl. Inter-assay coefﬁcients of variation were 3.5% at 0.68ng/dl and 3.3% at 1.64ng/dl for FT4. For FT3, they were 2.8% at 1.86pg/ml and 2.7% at 2.51pg/ml. Serum TSH was assayed by immune-radiometric assay method (TurboTax [125I] IRMA kit, Institute of Isotopes Co., Ltd. (Izotop), Hungary); the sensitivity of the assay was 0.011mIU/l.

### Statistical analysis

The analysis was undertaken using SPSS 16.0 software (IBM Corp.). Descriptive statistics have been expressed as the mean ± standard deviation (SD). Normality was checked using the Kolmogorov-Smirnov test. Correlation between variables was assessed using Pearson’s correlation analysis. The differences between the groups were analyzed using the independent sample t-test. Values of *p*<0.05 are considered statistically significant.

## RESULTS

### Characteristics of the participants

**Figure 1:**
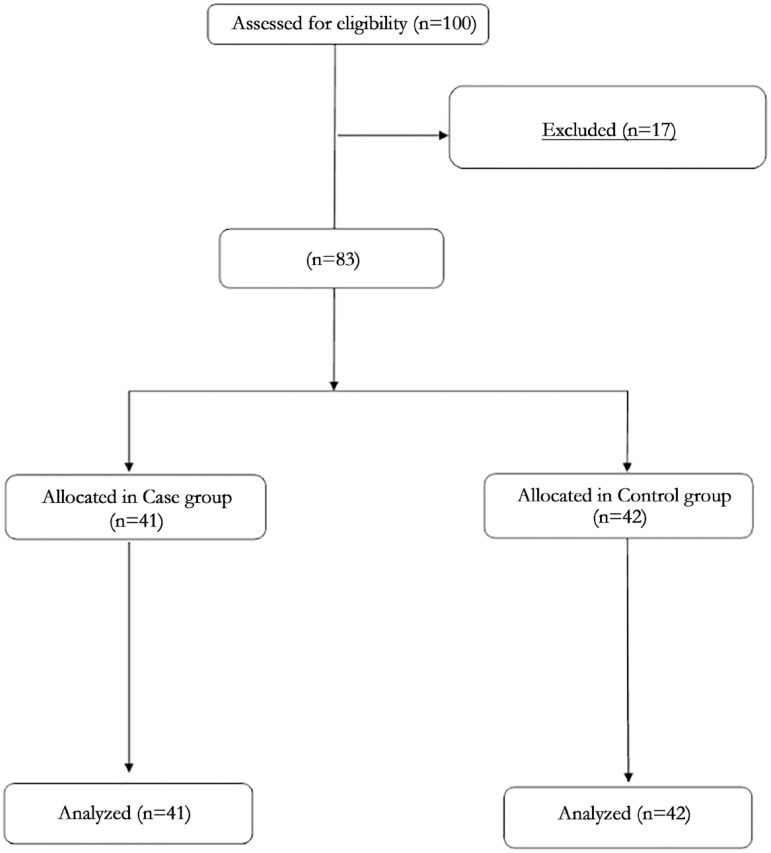
The summary of participants’ flow chart.

Seventeen of the one hundred individuals were excluded due to a lack of engagement. The eighty- three remaining participants (with a mean age of 20±2 years) completed the study. Figure 1 demonstrates the outcome of the study. The group with low quality sleep consisted of forty-one participants. There were forty-two individuals who had good sleep quality and hygiene. Both groups of individuals were well of similar age, were of the same sex and body mass index (BMI). The variables were normally distributed in this study (Table 1). The mean sleep score for individuals suffering from low quality sleep was 8.53 (±2.52) and for those with a normal quality of sleep, it was lower at 3.51 (±1.38) (Table 2).

**Table 1.** Characteristics of the participants.

	Low quality sleep group	Normal sleep group	*p* -value
Age (years)	20±2	20±2	NS *
Sex	Male	Male	NS
BMI (kg/m^2^)	24.1 ± 1.7	23.8 ± 1.5	NS

Values are means ± SD;

* NS: Non-signiﬁcant.

**Table 2.** Change in sleep score and stress score in the both of groups.

	Low quality sleep group	Normal sleep group	p-value
Sleep score	8.53±2.52	3.51±1.38	<0.001
Stress score	11.23±3.64	6.23±3.45	<0.001

Values are means ± SD; *p*-value<0.001: signiﬁcant.

### Thyroid function

The thyroid function test for all individuals suffering from poor quality sleep showed a higher serum concentration. The level of free T_3_ did not show a significant difference between the two groups. The free T_4_ level and TSH levels showed significant differences (*p*<0.05), compared with the individuals enjoying good sleep (Table 3).

**Table 3.** Change in FT_3_, FT_4_ and TSH hormones in the case and control groups.

Test	Low quality sleep group	Normal sleep group	p-value
FT_3_ (pg/ml)	3.43±0.51	3.24±0.38	0.09
FT_4_ (ng/dl)	1.56±0.22	1.41±0.23	0.01*
TSH (µIU/ml)	2.50±0.82	2.04±0.74	0.02*

Values are means ± SD; *p-*value<0.05: signiﬁcant.

### Correlations

Based on Pearson’s Correlation Test (Table 4) there is a significant correlation of serum FT4 values with sleep and stress score parameters in the low quality sleep group.

**Table 4.** Correlations between sleep score and stress score, and stress score and FT4 in the case group.

Parameters	Pearson correlation (r)	p-value
Sleep score and stress score	0.472	0.008
Stress score and FT_4_	0.384	0.03

Values are means ± SD; p-value<0.05: signiﬁcant.

## DISCUSSION

This study has investigated the relationship between thyroid function and sleep quality. It has shown that there is a correlation between thyroid hormones and thyroid-stimulating hormone levels relative to sleep quality and the level of stress suffered by the individual. Sleep restriction could have affected cognitive performance and other functions^21^.

Several animal model studies have found links between sleep and thyroid function^22,23^. Mullington et al.^24^ suggested that sleep deprivation can be a risk factor for the development of the cardiovascular disease. They suggested future research was needed to investigate the relationship between hormonal changes and sleep deprivation. Sathyanarayana et al.^25^ found that nightshift workers are at risk of increased TSH and sub-clinical hypothyroidism. Kuhs et al.^26^ found an elevation in the level of T3, T4, and TSH in individuals suffering from poor quality sleep. Balzano et al.^27^, suggested that the increase in energy expenditure during sleep deprivation of rats is at least in part mediated through enhanced brown adipose tissue (BAT) thermogenesis; induced by thyroid hormone as well as sympathetic stimulation. Jauch-Chara et al.^28^ indicated that an up-regulation of pituitary-thyroid activity, after short-term total sleep deprivation, led to an increase in TSH levels. Kuetting et al.^29^ found that sleep deprivation significantly increases cardiac contractility, blood pressure, and stress hormone secretion.

In comparison, there is a rise in serum TSH and thyroid hormone concentration following acute short sleep restriction. Kessler et al.^14^ mentioned that recurrent sleep restriction, in line with the short sleep times of a growing number of people in everyday life, can affect the function of the human thyroid axis. They demonstrated partial sleep loss was accompanied by modest but statistically significant declines in TSH and free T_4_, which were seen mainly in females. Based on their findings, rapid eye movement (REM) sleep deprivation provokes central hypothyroidism, which decreases TSH release and circulating T4 levels^30^.

In a large study of the population by Song et al.^16^, they suggested a relationship between subclinical hypothyroidism and poor sleep quality. Feng et al.^31^ demonstrated that there is no significant change in thyroid function in obstructive sleep apnea-hypopnea syndrome (OSAHS) in children before and after endoscopic adenoidectomy.

In conclusion, the current study shows that thyroid function (T4 and TSH) is significantly higher in those individuals suffering from poor sleep. The study has found correlations between sleep score, stress score and FT_4_ in this study group. This suggests sleep quality and stress levels can affect thyroid function. The mechanisms of sleep quality changes in human thyroid function have not been clarified. The data available in other research and these results about the association of thyroid hormone with sleep quality means there is justification for further investigations into this field to obtain more definitive results.
